# Normalize the response of EPID in pursuit of linear accelerator dosimetry standardization

**DOI:** 10.1002/acm2.12222

**Published:** 2017-11-10

**Authors:** Bin Cai, S. Murty Goddu, Sridhar Yaddanapudi, Douglas Caruthers, Jie Wen, Camille Noel, Sasa Mutic, Baozhou Sun

**Affiliations:** ^1^ Department of Radiation Oncology Washington University St. Louis MO USA; ^2^ Department of Radiation Oncology University of Iowa Hospitals and Clinics Iowa City IA USA; ^3^ Mallinckrodt Institute of Radiology Washington University School of Medicine St. Louis MO USA; ^4^ Varian Medical Systems Palo Alto CA USA

**Keywords:** EPID, pixel sensitivity map, quality assurance

## Abstract

Normalize the response of electronic portal imaging device (EPID) is the first step toward an EPID‐based standardization of Linear Accelerator (linac) dosimetry quality assurance. In this study, we described an approach to generate two‐dimensional (2D) pixel sensitivity maps (PSM) for EPIDs response normalization utilizing an alternative beam and dark‐field (ABDF) image acquisition technique and large overlapping field irradiations. The automated image acquisition was performed by XML‐controlled machine operation and the PSM was generated based on a recursive calculation algorithm for Varian linacs equipped with aS1000 and aS1200 imager panels. Cross‐comparisons of normalized beam profiles and 1.5%/1.5 mm 1D Gamma analysis was adopted to quantify the improvement of beam profile matching before and after PSM corrections. PSMs were derived for both photon (6, 10, 15 MV) and electron (6, 20 MeV) beams via proposed method. The PSM‐corrected images reproduced a horn‐shaped profile for photon beams and a relative uniform profiles for electrons. For dosimetrically matched linacs equipped with aS1000 panels, PSM‐corrected images showed increased 1D‐Gamma passing rates for all energies, with an average 10.5% improvement for crossline and 37% for inline beam profiles. Similar improvements in the phantom study were observed with a maximum improvement of 32% for 15 MV and 22% for 20 MeV. The PSM value showed no significant change for all energies over a 3‐month period. In conclusion, the proposed approach correct EPID response for both aS1000 and aS1200 panels. This strategy enables the possibility to standardize linac dosimetry QA and to benchmark linac performance utilizing EPID as the common detector.

## 1 INTRODUCTION

The use of electronic portal imaging device (EPID) has been investigated extensively over the past decade in quality assurance (QA) of linear accelerators (linac) and patient dosimetry.^1^, ^2^, ^3^, ^4^, ^5^, ^6^, ^7^ More recently, it has been proposed to use EPID for rapid linac acceptance test and linac daily QA.^8^, ^9^ Due to the convenient set‐up, high spatial resolution and availability on modern linacs, there has been an increased interest for EPID to be used as the common detector to standardize dosimetry measurements across different linacs. However, several challenges need to be resolved for accurate and reliable dosimetric measurements. Two major challenges were widely reported: the image lag (or ghosting effect),^10^, ^11^ and the difference in response of individual pixels of EPID panels.^12^, ^13^, ^14^ To use EPID as a common QA tool to benchmark and evaluate the linac dosimetry, the panel response differences must be neutralized.

One solution to normalize EPID response is to generate the pixel sensitivity map (PSM), which stores the relative gain correction factor for each pixel and is applied to correct the raw images. Several PSM generation approaches have been proposed and evaluated including (a) the Flood Field correction (FF) method^12^; (b) EPID with field horn‐removing add‐on phantom measurements^13^, ^14^; and (c) the Multiple small overlapping fields or continuous stripe fields method.^12^, ^13^ Among these approaches, manufacturer provided FF calibration is a simple built‐in approach to account for pixel response variability. However, the FF method removes the beam profile information which is the targeted feature for linac dosimetry QA. The add‐on phantom method utilizes a specially designed solid water phantom placed on top of EPID to remove the horn shape in the beam profile. The resultant relatively uniform/flat beam passing through the phantom is used to irradiate EPID and to derive the PSM. This approach requires extra effort for phantom design which depends on the radiation beam. The approach with multiple overlapping fields calibrates the pixel response utilizing overlapping small fields, e.g., 10 × 10 cm^2^, and irradiates the imager with the panel shifted to various locations. Since the supporting arm can induce backscattering (e.g., Varian aS500, aS1000 EPID panel), this method might not be accurate due to the backscatter change caused by large shifts of the imager. It has been reported that the backscatter generated from the EPID support arm could contribute up to 6% of maximum signal detected.^15^ To avoid backscatter from the imager arm, an approach was proposed^12^ using a set of 10 × 25 cm^2^ beam stripes to irradiate the panel while moving EPID only laterally (where backscatter was uniform). But this approach can only generate a one dimensional PSM in the lateral direction. In addition, the image lag or ghosting effect during image acquisition needs to be corrected to get an accurate PSM. The image lag has two effects: ghosting (the residual signal observed after radiation has ceased), and the signal increase for pixels that are continuously irradiated (which yields 4%–6% difference if not corrected^10^, ^11^).These two effects need to be eliminated or modeled to get the true pixel response, this is especially true for the overlapping field approach. After PSM correction, the previous reported stripe‐pattern or banding artifacts should be removed,^16^, ^17^ arm induced backscatter need to be corrected and the beam profile information should be preserved.

To derive a practical and efficient calibration method for generating a 2D PSM for a clinically configured EPID, several conditions need to be satisfied: (a) beam profile information needs to be preserved after correction; (b) large shifts of the panel need to be avoided due to backscatters induced by imager arm; (c) beam‐specific phantom design and build‐up setup should not be required; (d) image lag needs to be considered and (e) should be a rapid and convenient process for repeated clinical use. It has been reported that wide‐field array calibrations can be used to normalize detector's response.^18^ Recently, an approach utilizing large‐overlapping‐field irradiations with small imager shifts was proposed^19^ and showed promising results on an Elekta linac with the iViewGT EPID panel. In this study, we described a similar and improved large‐overlapping‐field algorithm utilizing an alternating beam and dark field technique (ABDF) and applied this technique to Varian linacs equipped with aS1000 and aS1200 EPID panels for photon and electron beams. The novelty of the proposed 2D PSM generation approach includes (a) adoption of the ABDF technique to eliminate image lag and maintain stable dose for each imaging frame; (b) development of XML‐scripts to automate the entire imaging acquisition process to improve efficiency; (c) first‐time derivation of electron beam PSM on Varian aS1000 EPID imager panel and both the photon and electron PSMs on the aS1200 EPID panel.

## 2 METHODS

### 2.A Equipment and EPID models

Linacs (TrueBeam, Varian Medical System, Palo Alto, CA, USA) equipped with either aS1000 or aS1200 amorphous silicon EPIDs were tested in this study. The aS1000 EPID model has a 40 × 30 cm^2^ active detector with 1024 × 768 pixels (spatial resolution 0.039 cm). The aS1200 EPID model has a larger active detector area of 43 × 43 cm^2^ with 1280 × 1280 pixels (spatial resolution 0.035 cm). The aS1200 model was engineered with a lead layer between the detector and the support arm to shield the arm induced backscatter; the aS1000 model does not have such shielding. Beam profiles in water were obtained using the Blue Phantom2 3D scanning system (IBA Dosimetry, GmbH, Germany) for this study.

### 2.B Image acquisition

The principal concept in derivation of a PSM presented here is to deliver several sets of large‐overlapping‐field irradiations to the EPID with small EPID shifts between each irradiation. Five sets of images were obtained with the panel at five discrete positions in a sequential order. The first set of images was acquired with the EPID at the center location with respect to the radiation beam. The other four sets of images were acquired with the EPID shifted left‐and‐right in lateral direction and toward‐and‐away in the gantry‐table direction. Each shift was 4 mm (approximately 10 pixels for aS1000 model and 12 pixels for aS1200 model). The source to imager distance (SID) was kept at 108 cm for all the image acquisitions. XML‐scripts (Varian TrueBeam Developer Mode 2.0) were developed to define the imaging acquisition mode named “ABDF technique” that automated the entire acquisition process. At each panel location, beam‐on MV images and beam‐hold dark field images were alternatively acquired until a total of 150 MUs were delivered. During the beam‐on time, 1.5 MUs were delivered for each beam‐on image with modulated dose rate and synchronized acquisition to ensure that the maximized signal was derived without saturating the imager. The dark fields taken during beam‐hold period were later subtracted from the raw images to eliminate the background noise and residual signal when radiation has ceased. The advantage of the ABDF technique is to eliminate the previously reported ghosting effects^10^, ^11^ for each frame and therefore reproduce the true pixel signal per frame. To demonstrate this process, a 25 cm × 25 cm field delivered use ABDF technique were shown in Fig. S1. The beam hold image (dark field) taken between beams showed a clear residual signal pattern. In this cases, the maximum residual signal intensity (~300) is around 1%–2% of the beam on peak intensity (~16000). This agrees with reference 10, 11. The raw images were then corrected by subtract the dark fields. A total of 10000 images were acquired within 4 min at the five positions. To avoid irradiation of the EPID electronics, the field size used was 27 × 37 cm^2^ for aS1000 model and 38 × 38 cm^2^ for aS1200 model.

The ABDF delivery technique defined here is not only used to synchronize the beam delivery and image acquisition but also to ensure no signal lose which is achieved by using a modulated dose rate. During the 1.5 MU delivery, the dose rate is varied to ensure the same amount of MU is delivered and received by the EPID panel. Moreover, a total of 150 MU is delivered at each location which results in 100 beam‐on images. During image postprocessing, the first 30 images were ignored to avoid beam instability, the last 70 images were averaged to reduce the output variations. In our initial testing, we tried 10, 30, 50, 70, 100, 150 and 200 images and found out that the output fluctuation was reduced and remained stable when averaged more than 50 images (75 MU). Therefore, the 150 MU (100 images or frames) were chosen and 70 images were averaged and used for PSM generation considering both fluctuation reduction and beam delivery efficiency.

### 2.C PSM Generation

Software programs (Matlab, The Mathworks INC., Natick, MA, USA) were developed for post image processing and PSM calculation. The alternating dark fields were first subtracted from the raw images for each frame, and then averaged out at each location. Bad pixel detection and image smoothing algorithms were also applied. The final five‐processed images, one at each location, were then used to calculate the PSM.

The details of the recursive algorithms used to derive the PSM has been discussed in Ref. 19, 20. We briefly summarized the process and key mathematical formulas here. Five set of EPID images were obtained. Image set obtained at center is labeled as (0,0) and is the reference for the other four sets of images with 10‐pixel shifts for the aS1000 model: left (−10,0), right (+10,0), superior (0,+10), and inferior (0,−10); or 12‐pixel shifts for the aS1200 model: left (−12,0), right (+12,0), superior (0,+12), and inferior (0,−12). Other quantities used in the algorithm: $F\left(i,j\right)$ is the fluence from the linac; *G*(*i*,* j*) is the gain factor map (or the PSM) of EPID relative to the central pixel; $I\left(i,j\right)$ is the final read‐out from EPID. Thus,

(1) $$I\left(i,j\right)=F\left(i,j\right)\times G\left(i,j\right)$$

For the center image,

(2) $$I_{C}\left(i,j\right)=F_{C}\left(i,j\right)\times G\left(i,j\right)$$

For the right image with a 10 pixel shift,

(3) $$I_{R}\left(i+10,j\right)=F_{R}\left(i+10,j\right)\times G\left(i+10,j\right).$$

Assuming that the averaged fluence delivered from the machine does not change, then

(4) $$F_{C}\left(i,j\right)=F_{R}\left(i+10,j\right)$$

By applying eq. (4) to eqs. (2) and (3),

(5) $$G\left(i+10,j\right)=\frac{I_{R}\left(i+10,j\right)}{Ic(i,j)}\times G\left(i,j\right)$$

for i=1,2,3,..,*N* where *N* is the number of pixles in direction *i*.

By assigning the central value $G\left(1,1\right)=1$, the other pixel gain factors can be calculated via recursively repeating this calculation. The 2D PSM can be similarly obtained for the other directions.

Following this approach, the 2D PSM for photon beams (6, 10 and 15 MV) and electron beams (6 and 20 MeV) were generated for both aS1000 and aS1200 imager panels. After derivation of the 2D PSM, the raw images can be corrected using the following formula

(6) $$I_{Corr}\left(i,j\right)=I_{Raw}\left(i,j\right)/G\left(i,j\right).$$

### 2.D Validations

A set of validation measurements were performed to evaluate the proposed method.

#### 2.D.1 Normalize EPID response across three dosimetrically matched linacs

Three linacs at the same institution were tuned to dosimetrically match each other; matching was verified by in‐water beam profiles. These in‐water beam profiles were measured using the Blue Phantom 2 3D scanning system (IBA Dosimetry, GmbH, Germany) and compared at the depth of maximum dose (D_max_) for photon beams and at reference depth (D_ref_) for electron beams. PSMs obtained on each linac were then used to normalize the raw EPID images. Because the beam profile measurements in water were matched to each other, the normalized EPID results were expected to match as well. To quantify the improvement after PSM correction, the maximum and mean percent differences and the 1D Gamma analysis with 1.5 mm, 1.5% criteria were performed for all energies tested.^21^, ^22^, ^23^

#### 2.D.2 Same phantom irradiation

The above validation tests were designed to test open field EPID images. In this section, two in‐house designed phantoms were used for EPID measurements and cross compared between two dosimetrically matched linacs. Two generic phantoms, one for photons and one for electrons, were used with the same set up on the two linacs with the aS1000 panel. The photon phantom was constructed on a water equivalent plastic (Solid Water, Gammex RMI) step wedge varying from 1 to 5 cm thickness, and with a testing logo attached at the center. The electron phantom was made with the testing logo placed at center of a 1 cm thick solid water slab with 1 cm thick rectangular stripes placed to the right. Radiation fields were delivered and images were acquired with the same beam settings on linacs 1 and 2 with the highest photon energy (15 MV) and the highest electron energy (20 MeV) available clinically.

#### 2.D.3 Short‐term reproducibility of the PSM

To evaluate the reproducibility of the PSM, two acquisitions 3 months apart were performed on linacs 1 and 2 mentioned above for all energies. The difference of pixel correction gain factor was analyzed.

## 3 RESULTS

### 3.A PSMs on aS1000 and aS1200 model panels

**Figure 1:**
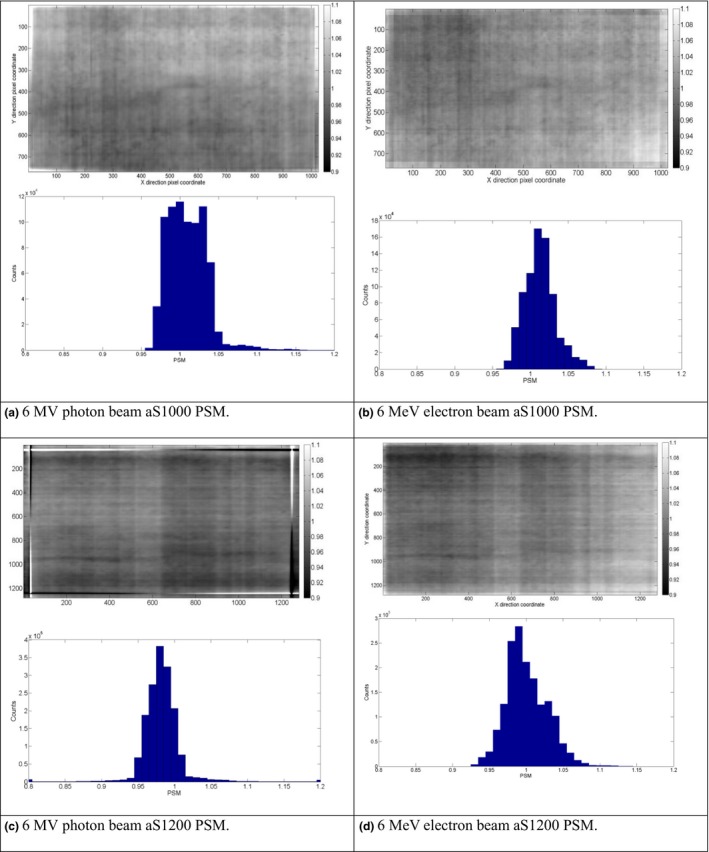
Derived PSMs and histogram statistics for 6 MV and 6 MeV beams of aS1000 and aS1200 models. (a) 6 MV photon beam aS1000 PSM.(b) 6 MeV electron beam aS1000 PSM. (c) 6 MV photon beam aS1200 PSM. (d) 6 MeV electron beam aS1200 PSM.

Figure 1 shows the derived 2D PSM array and histograms of pixel value for 6 MV photon beams and 6 MeV electron beams on the aS1000 and aS1200 panels. The majority of pixel gain correction factors were in the range from 0.9 to 1.1. A value of 1.0 indicated no correction was needed, a value >1.0 indicated over response, and a value <1.0 indicated under response. Longitudinally (gantry‐couch direction) oriented stripe‐patterns can be observed for all PSMs due to the line readout mechanism. For the aS1000 model [Figs. 1(a) and 1(b)], the photon PSM revealed that more pixels with >1.0 gain correction factor presented at the gantry side (Y coordinates with the lower values) compared to the pixels near the couch side (Y coordinates with the higher values). This was due to the arm‐induced backscatter which mainly concentrates at the gantry side of the imager. For electrons, this effect was not obvious due to less scatter originating from the arm, which resulted in a relatively narrower histogram. Compared to the aS1000 model, the PSM histogram was more centralized for the aS1200 model [Figs. 1(c) and 1(d)] due to the backscatter shielding. Similar patterns were observed on the PSM for 10 MV, 15 MV, and 20 MeV PSM generation. The relative gain factor distribution showed that the PSM is dependent on beam energies and beam modalities, which implied that it is necessary to generate and apply the PSM for various energy photon and electron beams separately.

### 3.B PSM corrected and uncorrected beam profile comparison

**Figure 2:**
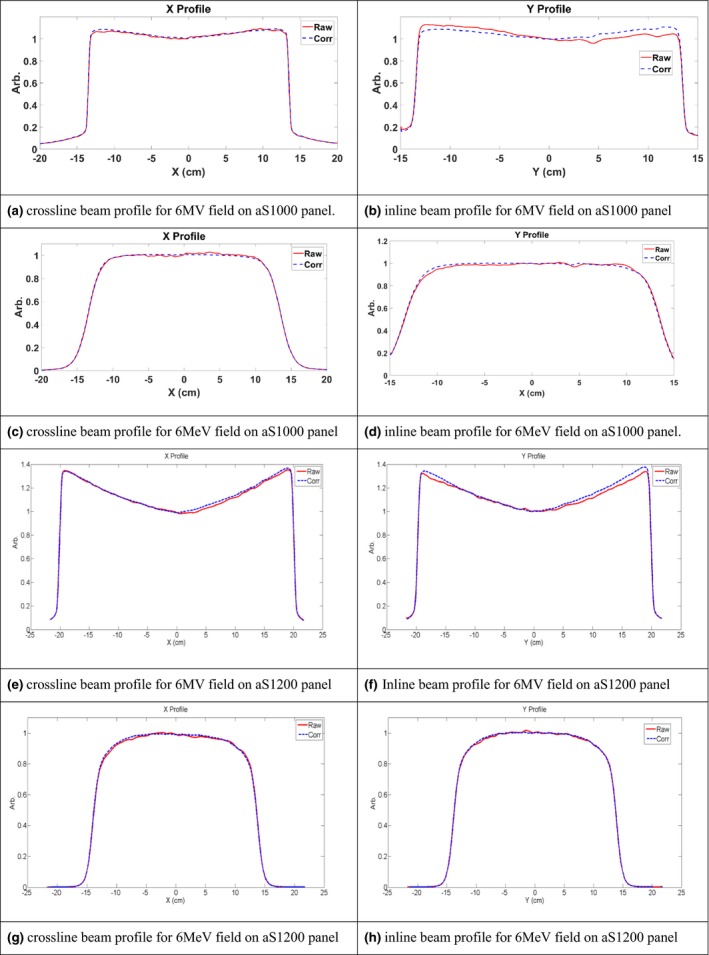
EPID image beam profiles of 6 MV and 6 MeV fields before and after PSM correction on aS1000 and aS1200 panels. 6 MV field on aS1000 panel crossline (a) and inline (b) beam profiles on aS1000 panel. 6 MeV field on aS1000 panel crossline (c) and inline (d) beam profiles. 6 MV field on aS1200 panel crossline (e) and inline (e) beam profiles. 6 MeV field on aS1200 panel crossline (g) and inline (h) beam profiles.

For the aS1000 panel, raw and PSM corrected EPID measurements of beam profiles for 6 MV photon fields and 6 MeV electron fields at a field size of 25 × 25 cm^2^ are presented in Figs. 2(a)–2(d). For the photon field, the stripe patterns and increased intensity for pixels near the gantry side were observed in the raw images. The PSM normalization corrected both effects, and the flattening filter pattern was reproduced after correction. Beam profiles in the Y direction (gantry‐couch) in raw images were asymmetrical due to the backscatter from the imager arm. The postcorrection Y profile was more symmetric. The X beam profile in the raw image was relatively symmetrical due to the uniform backscatter in this direction, but showed “wiggling” due to the stripe pattern from the line readout mechanism. The PSM corrects this artifact and smooths out the beam profile. For electron beams, the PSM further eliminated the stripe pattern in the raw images and the corrected images show a more uniform intensity distribution across the panel.

Regarding the aS1200 panel, the raw and PSM corrected EPID measurement of beam profiles for 6 MV photon fields with a field size of 40 × 40 cm^2^ and for 6 MeV electron fields with a field size of 25 × 25 cm^2^ are presented in Figs. 2(e)–2(h). Similar to the aS1000 panel, the stripe patterns were observed in the raw image and eliminated after PSM correction in the photon beams. Both the X and Y beam profiles showed a more symmetrical shape due to the backscatter shielding compared to aS1000 panel. Also, similar to the aS1000 panel, for electron beams, the PSM corrected the stripe patterns and the beam profiles were more uniform after correction. Similar behavior was observed for other energies on both panels.

### 3.C PSM normalized EPID response across three linacs with matched dosimetry

In this section, we tested the hypothesis that PSM normalized EPID measurements can be used to standardize linac dosimetry and to benchmark machine performance. Superimposing the in‐water beam profiles and percent depth dose (PDD) curves for the three linacs tested demonstrates they are closely matched. We show that the beam profiles derived from the EPID measurement on these linacs were matched after the PSM normalization.

**Figure 3:**
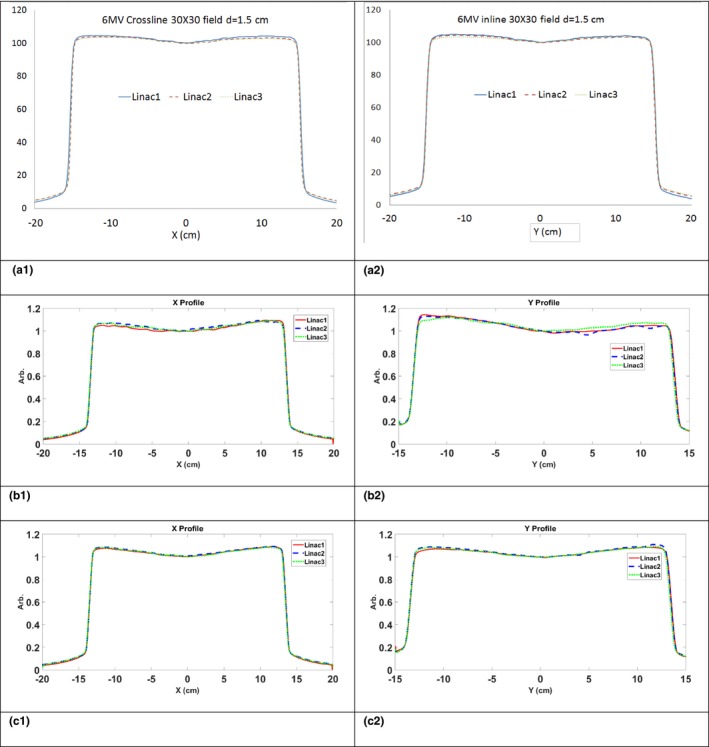
6 MV beam profiles. Top row: in water measurement crossline (a1)) and inline (a2) beam profiles. Middle row: EPID measurement without PSM normalization for crossline (b1) and inline (b2) beam profiles. Bottom row: EPID measurement after PSM normalization crossline (c1) and inline (c2) beam profiles.

**Figure 4:**
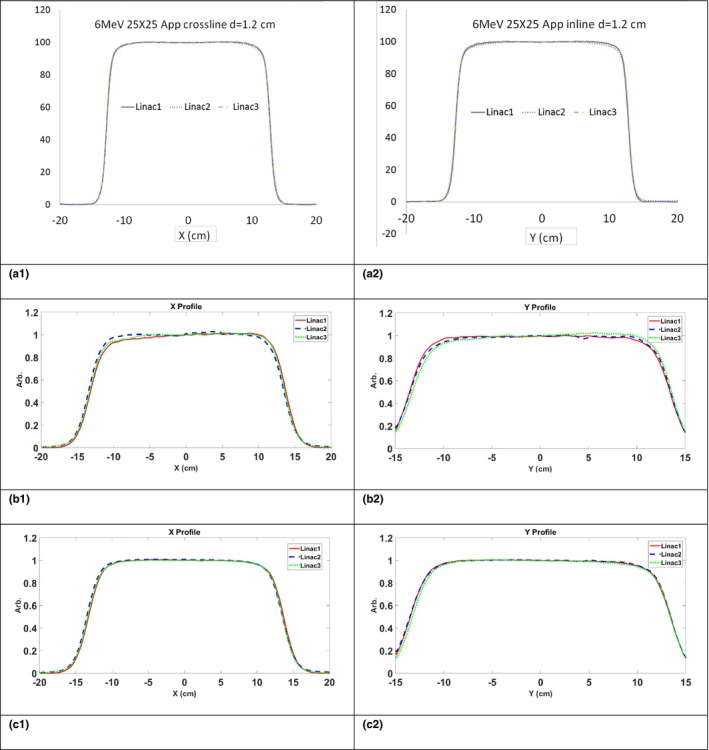
6 MeV beam profiles. Top row: in water measurement crossline (a1)) and inline (a2) beam profiles. Middle row: EPID measurement without PSM normalization for crossline (b1) and inline (b2) beam profiles. Bottom row: EPID measurement after PSM normalization crossline (c1) and inline (c2) beam profiles.

In Figs. 3 and 4, 6 MV photon beams and 6 MeV electron beams, crossline (X) and inline (Y) in‐water beam profiles are shown in Figs. 3a(1) and 3a(2) for three linacs that were equipped with aS1000 imager panel. The beam profiles without PSM normalization are shown in Figs. 3b(1) and 3b(2). The PSM corrected beam profiles are shown in Figs. 3c(1) and 3c(2). Though the water scans are matched, without correction, the raw beam profiles did not match across the three machines. “Wiggling” in the beam profiles due to stripe patterns mentioned previously, and the backscatter‐induced asymmetry were present in the raw images. After PSM correction, the beam profile matched much closer to each other for both photon and electron fields. The stripe pattern artifact and the arm‐induced over response was corrected.

The max and mean percent differences between beam profiles before and after PSM corrections were listed in Table 1. Both maximum and average percent differences were reduced after PSM correction for all energies indicating a better beam matching is achieved after PSM correction. The results of 1D Gamma tests are listed in Table 2. The crossline beam profiles had an average 10.5% improvement in the Gamma passing rate and a 26% maximum improvement. The inline beam profile had an average of 37% improvement and a 44% maximum improvement. The improvement in beam profile matching observed for inline profiles indicates that the PSM successfully corrected the arm‐induced backscatter.

**Table 1:** Maximum and average percent difference comparisons of matched beam profiles

Energy	Beam profile difference (linac 1 vs linac 2)								Beam profile difference (linac 1 vs linac 3)							
	Crossline				Inline				Crossline				Inline			
	Precorrection		Postcorrection		Precorrection		Postcorrection		Precorrection		Postcorrection		Precorrection		Postcorrection	
	Max %	Mean %	Max %	Mean %	Max %	Mean %	Max %	Mean %	Max %	Mean %	Max %	Mean %	Max %	Mean %	Max %	Mean %
6 MV	3.0	1.5	1.0	0.5	3.0	0.6	1.4	0.4	2.4	0.8	0.8	0.3	2.8	1.6	0.7	0.3
10 MV	3.1	1.7	1.5	0.7	4.3	1.1	1.4	0.6	2.4	0.8	1.5	0.4	2.4	1.0	1.4	0.6
6 MeV	4.7	1.7	1.1	0.4	2.9	0.9	0.9	0.3	2.7	0.8	0.9	0.3	4.2	1.9	1.2	0.4
20 MeV	3.3	1.7	2.5	0.9	3.1	0.6	1.1	0.4	3.0	1.0	1.5	0.3	2.7	1.4	1.1	0.4

**Table 2:** Gamma passing rate comparisons of matched beam profiles

Energy	Gamma Analysis (linac 1 vs linac 2)				Gamma Analysis (linac 1 vs linac 3)			
	Crossline		Inline		Crossline		Inline	
	Precorrection	Postcorrection	Precorrection	Postcorrection	Precorrection	Postcorrection	Precorrection	Postcorrection
6 MV	89%	99%	53%	96%	95%	99%	56%	93%
10 MV	93%	99%	85%	95%	92%	92%	58%	93%
6 MeV	83%	98%	35%	86%	70%	96%	52%	82%
20 MeV	74%	95%	47%	96%	94%	96%	48%	92%

### 3.D Irradiations on two linacs with the same phantom

**Figure 5:**
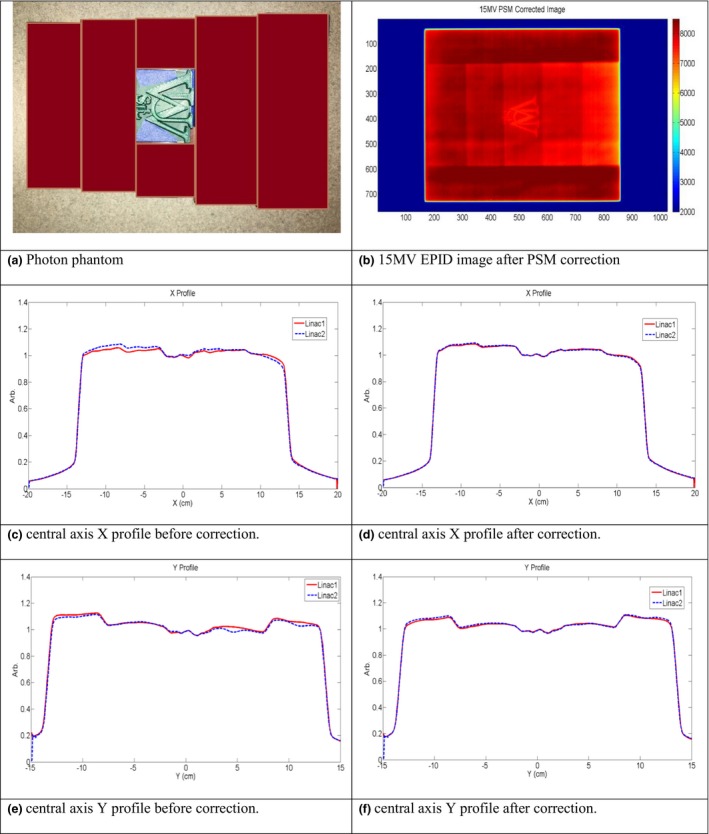
(a) Photon phantom used. (b) 15 MV EPID image after PSM correction. (c) Central axis crossline beam profile before correction. (d) Central axis crossline beam profile after correction. (e) Central axis inline beam profile before correction. (f) Central axis inline beam profile after correction.

**Figure 6:**
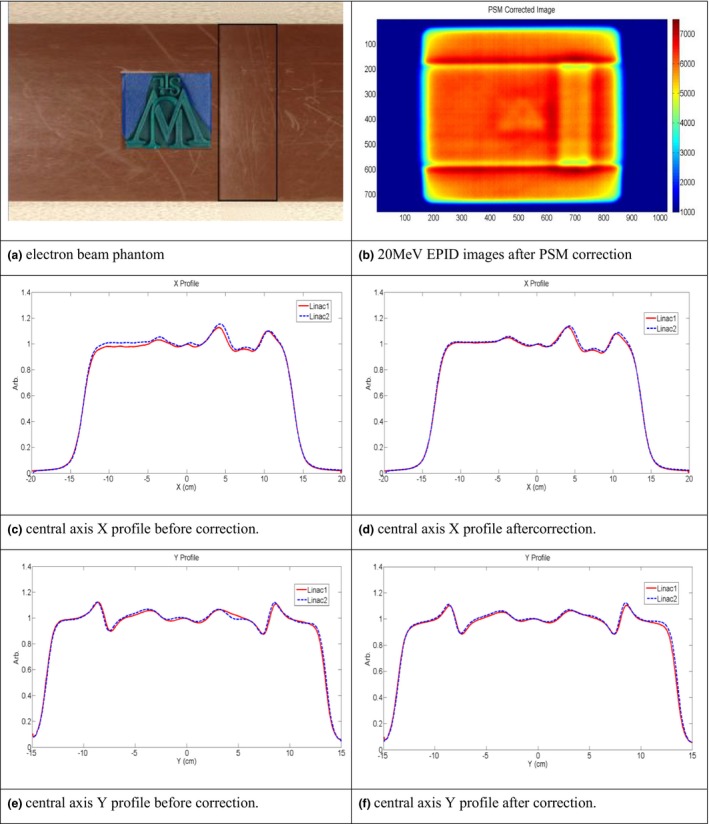
(a) Electron phantom used. (b) 20 MeV EPID images after PSM correction. (c) Central axis crossline beam profile before correction. (d) Central axis crossline beam profile after correction. (e) Central axis inline beam profile before correction. (f) Central axis inline beam profile after correction.

Two phantoms, one designed for photon measurements and one for electron measurements, are shown in Figs. 5 and 6. The same setup was used for each phantom irradiation on linac1 and linac2.

For the photon fields, the varying intensity of the beam after passing through the step wedges and the testing logo was apparent in the EPID images in both crossline (X) and inline (Y) direction. For electron fields, the fluctuation of beam intensity after passing through the base slab, the rectangular stripe and testing logo were also observed in these two directions. The raw beam profiles did not match as closely as the PSM corrected profiles. The maximum and average percent difference of the beam profiles were also both improved after PSM correction. For 15 MV, the maximum and average difference was reduced from 3.7% and 1.4% to 0.9% and 0.4% for crossline beam profile; from 4.0% and 1% to 1.4% and 0.5% for inline beam profile. For 20 MeV, the maximum and average difference reduced from 5.6% and 1.9% to 3.7% and 0.9% for crossline beam profile; from 4.3% and 1.1% to 3% and 0.9% for inline beam profile. The 1D gamma analyses of beam profiles indicated that the passing rating improved from 82% to 95% inline and from 71% to 99% crossline for the 15 MV photon beam; and 94% to 95% inline and 60% to 82% crossline for the 20 MeV electron beam. Similarly to the open field testing, these results demonstrate that the PSM normalization improves agreement between two dosimetry measurements on two linacs while preserving the native beam dosimetry features. These results also indicate that with high spatial resolution, the EPID measurements are able to detect subtle dosimetric changes or linac performance variations.

### 3.E Reproducibility of the PSM over a 3‐month period

**Figure 7:**
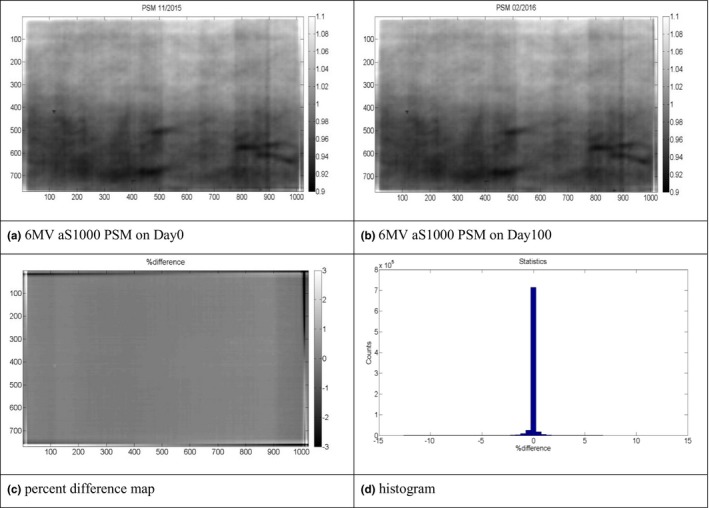
Two PSMs generated for 6 MV photon beams with 3 months apart. (a) 6 MV aS1000 PSM on Day0. (b) 6 MV aS1000 PSM on Day100. (c) Percent difference map. (d) Histogram.

During a 3‐month period, there was no major changes to the imager. The obtained PSM of 6 MV on Day0 and Day100 are plotted in Fig. 7. The 2D percent difference map and histogram showed that the majority of pixels have a less than 1% difference. Similar results were observed for other energies. This comparison demonstrates that the generated PSM could be repeatedly used over the time period as long as there is no major change to EPID for both photon and electron beams.

## 4 DISCUSSION

In this study, a self‐sufficient standardization strategy targeting linac dosimetry utilizing a PSM to normalize EPID response was introduced and validated. The proposed PSM generation approach is easy to execute and robust. Other work has discussed how to convert EPID measurements to in‐water measurements, and this could be accomplished with the PSM corrected images and then applying the off‐axis response correction, as presented by several groups previously^**.**^ 12, 23 However, the aim of this study is to normalize EPID response so it can be used as a common detector for machine performance benchmarking.

The implementation and validation of this method was conducted on two Varian EPID models (aS1000 and aS1200) with Varian TrueBeam linacs. The arm induced backscattering (asymmetry observed in radial (Y) beam profiles) is a challenge for measurements with aS1000 panels, especially for large fields which are frequently used for machine dosimetry QA. The proposed methods corrected the stripe pattern artifacts present in the raw images for both the aS1000 and aS1200 model panels, and further corrected the over‐response due to backscattering for the aS1000 panel. With ABDF, the residual signal was removed before each irradiation and the impact of output and beam profile fluctuation^18^, ^19^ was reduced via multiframe averaging. PSM normalization significantly improved the agreement between EPID measurements delivered on different machines with matched dosimetry in both the open field and the phantom study.

Some limitations worth noting are that the Varian developer mode was used as the platform for this study. Since the acquisition mode was a user defined imaging mode, XML‐scripts had to be used to drive the acquisition process. The efficiency will be significantly reduced if hundreds of images are acquired manually. Moreover, since the proposed method relies on the use of overlapping features to generate the PSM, the gain factors obtained for the nonoverlapping regions, such as the pixels near imager boundary, are not accurate. For the aS1200 model panel, this is not a limitation since the maximum 40 × 40 cm^2^ field would falls into the central region. But for aS1000 model panel, the maximum field size that could be accurately reconstructed in this study was limited to 27 × 37 cm^2^. Also, since the PSMs were derived using the full panel irradiation, the backscatter present, especially for aS1000 panel, was at a maximum. Therefore, the PSM tends to overcorrect the backscatter for smaller fields, especially at the field edge and for lower photon energy beams. The positional accuracy is critical for this calibration method. The current XML programming allows 1 mm digital sensibility while moving the EPID panel. According to Varian's technique guideline, the positioning accuracy of glass within imager and arm mounting is estimated to be <1 mm. Since the used beam shape has an intensity gradient of up to 0.2% per mm (at the border of the imager), a less than 1 mm shift would result in deviation up to 0.2%. For beam profile matching comparisons, we calculated the maximum and average differences and used the Gamma analysis as metrics to quantify the improvement. Though the gamma analysis has some limitations, it is a standard indicator and used by many publications (Ref. 21, 22) for beam profile comparisons.

## 5 CONCLUSION

The proposed strategy derives a PSM for both aS1000 and aS1200 model panels. The derived PSM can be used to normalize the EPID response and recreate the linac dosimetric features. This strategy enables the possibility to standardize measurements on different machines which would enable to benchmark the linac performance with the EPID used as the common detector and thereby reducing the dependency on third party QA tools.

## CONFLICT OF INTEREST

This project received funding from Varian Medical System.

## Supporting information


**Fig. S1**. A single frame obtained via ABDF technique. (a) beam on images (b) beam hold (dark field) (c) post correction image. A clear residual pattern can be seen on the beam onld image.
