# Supplementary figures and images for: Normalize the response of EPID in pursuit of linear accelerator dosimetry standardization
Source: J Appl Clin Med Phys. 2017 Nov 10;19(1):73–85. doi: 10.1002/acm2.12222 (PMC5768011; doi:10.1002/acm2.12222)

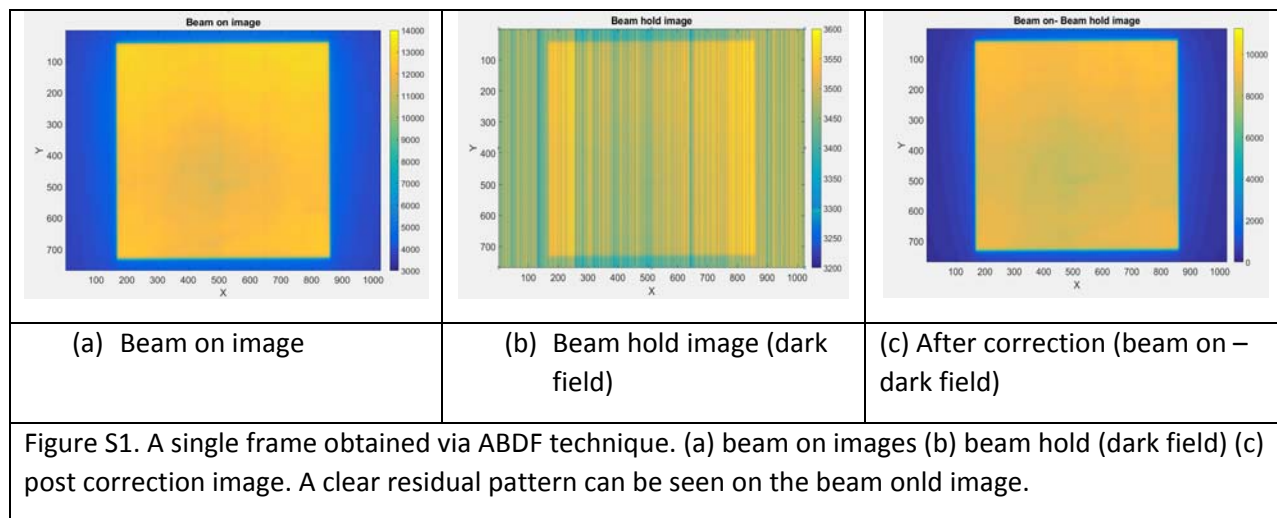

Supplement: Supplementary file 1 — Fig. S1. A single frame obtained via ABDF technique. (a) beam on images (b) beam hold (dark field) (c) post correction image. A clear residual pattern can be seen on the beam onld image. [file ACM2-19-73-s001.pdf]
